# Impact of CTLA-4 checkpoint antibodies on ligand binding and Transendocytosis

**DOI:** 10.3389/fimmu.2022.871802

**Published:** 2022-08-31

**Authors:** Cayman Williams, Alan Kennedy, Maximillian A. Robinson, Christopher Lloyd, Simon J. Dovedi, David M. Sansom

**Affiliations:** ^1^ University College London (UCL) Institute of Immunity and Transplantation, London, United Kingdom; ^2^ Biologics Engineering, R&D, AstraZeneca, Cambridge, United Kingdom; ^3^ Early Oncology R&D, AstraZeneca, Cambridge, United Kingdom

**Keywords:** CTLA4, checkpoint blockade, anti-CTLA4, transendocytosis, ipilimumab, tremelimumab

## Abstract

Anti-CTLA-4 antibodies have pioneered the field of tumour immunotherapy. However, despite impressive clinical response data, the mechanism by which anti-CTLA-4 antibodies work is still controversial. Two major checkpoint antibodies (ipilimumab and tremelimumab) have been trialled clinically. Both have high affinity binding to CTLA-4 and occupy the ligand binding site, however recently it has been suggested that in some settings such antibodies may not block ligand-CTLA-4 interactions. Here we evaluated blocking capabilities of these antibodies in a variety of settings using both soluble and cell bound target proteins. We found that when ligands (CD80 or CD86) were expressed on cells, soluble CTLA-4-Ig bound in line with affinity expectations and that this interaction was effectively disrupted by both ipilimumab and tremelimumab antibodies. Similarly, cellular CTLA-4 binding to soluble ligands was comparably prevented. We further tested the ability of these antibodies to block transendocytosis, whereby CTLA-4 captures ligands from target cells during a cognate cell-cell interaction. Once again ipilimumab and tremelimumab were similar in preventing removal of ligand by transendocytosis. Furthermore, even once transendocytosis was ongoing and cell contact was fully established, the addition of these antibodies could prevent further ligand transfer. Together these data indicate that the above checkpoint inhibitors performed in-line with predictions based on affinity and binding site data and are capable of blocking CTLA-4-ligand interactions in a wide range of settings tested.

## Background

CTLA-4 is an immune checkpoint, which functions to limit the activation of self-reactive T cells, which exist within the normal immune system. CTLA-4 is highly expressed on regulatory and activated T cells and binds to two different ligands, CD80 and CD86 with varying affinity. These same ligands are shared with an activating receptor, CD28. Accordingly, CD28 activation and CTLA-4 inhibition are intimately linked by their shared ligands (1, 2). Genetic defects in CTLA-4 function lead to profound and sometimes fatal autoimmunity, which is dependent on ligand-driven CD28 activity (3–5).

The above balance between T cell activation and inhibition has made the CTLA-4 pathway an attractive target for therapeutic intervention. Initially, the use of a soluble CTLA-4 molecule (e.g. Abatacept) was shown to be useful as an immunosuppressive agent, which blocks the availability of CD80 and CD86 ligands (6). This in turn impairs CD28 stimulation of T cells, suppressing T cell and (indirectly) B cell responses. In contrast, immune activating antibodies targeting CTLA-4 are used in cancer immunotherapy (7, 8). Here, preventing the normal function of CTLA-4 heightens activity within the T cell compartment, resulting in durable anti-tumour immune responses in a proportion of patients. However, a major feature of CTLA-4 inhibition is the considerable side-effect profile resulting from the activation of self-reactive T cells (9).

Given the therapeutic importance of the CTLA-4 pathway, efforts have been made to understand the mechanism of action of anti-CTLA-4 mAb such as ipilimumab and tremelimumab in anti-tumour responses. Several non-exclusive possibilities exist, including the blocking of CTLA-4 function on regulatory T cells, Fc-mediated depletion of regulatory T cells and effects on activated T cells directly (10–12). It has been widely assumed, given their binding characteristics, that whatever the mode of action, anti-CTLA-4 antibodies would block CTLA-4-ligand interactions. We have proposed that the ability of CTLA-4 to physically remove ligands from antigen presenting cells by transendocytosis is likely to be a key functional mechanism used by regulatory T cells (13, 14) and therefore ipilimumab and tremelimumab would be predicted to block transendocytosis. Surprisingly, it was recently reported that ipilimumab did not block CTLA-4-ligand interactions in some contexts (15), despite crystallographic studies indicating that both ipilimumab and tremelimumab overlap the CTLA-4 ligand binding site (16), suggesting that other constraints may exist.

We have therefore re-assessed anti-CTLA-4 mAbs for their ability to disrupt CTLA-4-ligand interactions in several different settings including transendocytosis between T cells and APCs. We observed that ipilimumab and tremelimumab were both capable of robustly blocking CTLA-4-ligand interactions using soluble reagents in settings where competition between ligands and antibody occurred. However, in situations where CTLA-4-ligand interactions were pre-established, we did not observe significant displacement by anti-CTLA-4 antibodies (particularly for CD80) indicating that pre-established CTLA-4-Ligand interactions are not readily disrupted by anti-CTLA-4. In contrast to previous reports, we also observed clear, dose dependent blockade of transendocytosis, where both ligand and receptor were expressed in their normal membrane settings. Furthermore, we observed that even once transendocytosis (and therefore cell contact) was established further ligand interactions could still be prevented by anti-CTLA-4 mAb. These observations were recapitulated across different cell types, indicating that they were not cell-type dependent. Taken together these data indicate that both ipilimumab and tremelimumab exert robust blockade of CTLA-4–ligand interactions in cellular contexts where competition for CTLA-4 between antibody and ligand binding can occur. Accordingly, the functional importance of ligand blockade vs. other mechanisms of anti-CTLA-4 mAb efficacy cannot be inferred from suggestions that ipilimumab, (and as also shown here tremelimumab) do not block CTLA-4 in some cellular contexts.

## Methods

### Generating CD80-GFP, 86 GFP and CTLA4 cell lines

Full length CTLA-4 cDNA, full length GFP-tagged CD80 or GFP-tagged CD86 were cloned into the pMP71 retroviral vector (17) using the NotI and RsrII restriction sites to generate pMP71-CTLA-4, CD80GFP or CD86GFP. Retroviral supernatants were obtained by transfection of Phoenix-Amphoteric packaging cells in combination with pVSV, using the FUGENE HD transfection reagent (Roche Molecular Biochemical). Retrovirus-containing supernatants were used to transduce Chinese hamster ovary (CHO) cells (CTLA-4, CD80GFP, CD86GFP), Jurkat cells (CTLA-4), or DG-75 B lymphocyte cells (CD80GFP or CD86GFP). Single-cell cultures of transduced CHO cells were produced by serial-dilution and expanded to generate clonal lines of CTLA-4 WT, CD80GFP or CD86GFP expressing CHO cells. Jurkat and DG-75 lines were generated by sorting on target populations using the BD FACSAria™ *Fusion* Flow Cytometer.

### Ig fusion proteins

CD80 (Cat# 10133-B1) and CD86 (Cat# 141-B2) -Ig expressing human IgG1 Fc were purchased from R&D Systems.

CTLA-4-Ig human IgG 1-Fc (Abatacept) was made by Bristol-Myers Squibb and purchased from the Royal Free Hospital pharmacy.

Fluorochrome conjugation was carried out using the PE/R-Phycoerythrin (Cat# ab102918) or APC (Cat# ab201807) conjugation kit - Lightning-link (Abcam) according to manufacturers instructions and the protein used at the concentrations stated in the figure legends.

### Ipilimumab and tremelimumab targeting of cell bound CTLA-4

To compare how anti-CTLA-4 Abs interact with cell bound CTLA-4 in the presence of soluble CD80 or CD86 ligand, we used Jurkat cells transduced with CTLA-4, soluble CD80/CD86-Ig and fluorochrome-conjugated anti-CTLA-4 Abs. 1x10^5^ Jurkat cells were plated per well in 96 U-bottom plates, CD80/CD86-Ig doses ranged between 50µg/ml and 0.0031µg/ml with 4-fold dilution intervals (including 0µg/ml) and anti-CTLA-4 Abs were used at 2µg/ml. As cell surface expressed CTLA4 undergoes rapid and constitutive internalisation, all reagents and cells were pre-chilled on ice to stabilise CTLA-4 at the surface and therefore allow us to investigate how anti-CTLA-4 Abs and soluble ligand interreact with a stable level of surface CTLA-4. In some cases, a CTLA-4 mutant with no cytoplasmic domain (CTLA-4 Del 36) was used to further ensure that CTLA-4 could not internalise. All wash buffers were also maintained at the temperature appropriate to the experiment (either 4°C or 37°C) as indicated. Binding of ipilimumab and tremelimumab to cells lacking CTLA-4 was used as a specificity control (**Figure S1**).

Where the ability of anti-CTLA-4 Abs to displace CTLA-4-ligand interactions was tested, Jurkat CTLA-4+ cells were stained with a titration of soluble CD80/CD86-Ig for 30 minutes on ice. Cells were washed 4 times with PBS and subsequently stained with PE conjugated anti-CTLA-4 for 30 minutes on ice. Cells were washed 4 times with PBS and cells analysed for anti-CTLA-4 staining by flow cytometry.

Where the ability of anti-CTLA-4 Abs to directly compete with soluble CD80/CD86-Ig was tested, PE conjugated anti-CTLA-4 was mixed with a titration of soluble CD80/CD86-Ig. This solution was added to Jurkat CTLA-4+ cells, incubated on ice for 30 minutes, washed 4 times with PBS and anti-CTLA-4 staining analysed by flow cytometry.

Where the ability of anti-CTLA-4 Abs to prevent soluble ligand binding to cell bound CTLA-4 was tested, Jurkat CTLA-4+ cells were stained with PE conjugated anti-CTLA-4 for 30 minutes on ice, washed 4 times with PBS and stained with a titration of soluble CD80/CD86-Ig at either 4°C or 37°C as stated in the legend. Cells were washed 4 times in PBS and analysed for anti-CTLA-4 staining by flow cytometry.

### Ipilimumab and tremelimumab targeting of soluble CTLA-4-Ig

To compare how anti-CTLA-4 Abs interact with soluble CTLA-4-Ig (Abatacept) in the presence of cell bound ligand, we used DG75 B cells transduced with GFP-tagged CD80 or CD86, in the presence of soluble fluorochrome-conjugated CTLA-4-Ig and anti-CTLA-4 Abs. For these experiments 1x10^5^ DG75 cells were plated per well in 96 U-well plates, CTLA-4-Ig was used at 2µg/ml and anti-CTLA-4 Ab titrated between 50µg/ml and 0.0031µg/ml with 4-fold dilution intervals (including 0µg/ml). All reagents, cell lines and wash buffers were maintained at the appropriate temperature (either 4°C or 37°C as stated in the legend). Non-binding human isotype control antibodies, IgG1 (ipilimumab control) or IgG2a (trememlimumab control) were sourced from AstraZeneca and BioXcell respectively and were used at 50µg/ml to establish specificity of CTLA-4 blockade (**Figure S1**).

Where the ability of anti-CTLA-4 Abs to block soluble CTLA-4-Ig from binding to cell bound CD80/CD86 was tested, soluble APC conjugated CTLA-4-Ig was mixed with a titration of anti-CTLA-4. This mixture was added to DG75 CD80 or CD86 cells, incubated on ice for 30 minutes, washed 4 times with PBS and CTLA-4-Ig staining analysed by flow cytometry.

Where the ability of anti-CTLA-4 Abs to directly compete with cell bound CD80/CD86 for soluble CTLA-4-Ig was tested, DG75 CD80 or CD86 cells were mixed with a titration of anti-CTLA-4 Abs before soluble APC conjugated CTLA-4-Ig was added. Cells were incubated on ice for 30 minutes, washed 4 times with PBS and analysed for CTLA-4-Ig staining by flow cytometry.

Where the ability of anti-CTLA-4 Abs to displace CTLA-4-Ig-ligand interactions was tested, DG75 CD80 or CD86 cells were stained with APC conjugated CTLA-4-Ig on ice for 30 minutes, washed 4 times with PBS and then treated with a titration of anti-CTLA-4 Abs on ice or at 37°C for 30 minutes. CTLA-4-Ig staining was measured by flow cytometry.

### Anti-CTLA4 blockade of CTLA-4-mediated transendocytosis of CD80 and CD86

CTLA-4 transduced CHO or Jurkat cells were cultured in the presence of CTV labelled CD80 or CD86-GFP transduced CHO or DG75 B cells at a ratio of 1 CTLA-4 expressing cell:1 ligand expressing cell for 5 hours at 37°C in 96 U-well plates. Anti-CTLA-4 Abs were added at the beginning of the assay at a dose ranging between 50µg/ml and 0.0031µg/ml with 4-fold dilution intervals (including 0µg/ml) and transendocytosis analysed by flow cytometry. Percentage ligand (GFP) loss by transendocytosis was measured by taking the GFP MFI of anti-CTLA-4 treated conditions relative to ligand GFP MFI in control samples where no CTLA-4 was present. Gating was performed on CTV +ve (ligand donor cells) or CTV-ve (CTLA-4 recipient cells) to calculate changes in loss of ligand from donor cells (CTV +ve) and uptake by CTLA-4 recipient cells (CTV -ve) cells. Example gating is shown in **Figure S2**.

### Anti-CTLA-4 blockade of pre-established transendocytosis of CD80 and CD86

CTLA-4 transduced CHO or Jurkat were cultured in the presence of CTV labelled CD80-GFP or CD86-GFP transduced CHO or DG75 B cells at a ratio of 1 CTLA-4 expressing cell:1 ligand expressing cell over time at 37°C in 96 U-well plates. Anti-CTLA-4 Abs were pipetted gently into the well to prevent disturbing pre-established cell-cell contacts at 0, 2.5, 3 or 6 hours post-culture. Transendocytosis was measured by flow cytometry and ligand loss quantified as stated above.

### Fitting dose response curves

Dose response curves were fitted and LogEC_50_ and LogIC_50_ were calculated using GraphPad Prism v6. Error bars on dose response curves represent Mean+/-SD, bar charts present Mean with 95% confidence intervals.

## Results

### Blocking of soluble CTLA-4-ligand interactions by checkpoint antibodies

It has been suggested that anti-CTLA-4 blocking activity may depend on whether CTLA-4 or ligand was in the bound or soluble phase. Given that ipilimumab and tremelimumab have been shown to have similar interactions and binding sites with CTLA-4 (16) that overlap with CD80 and CD86 binding we wanted to compare both antibodies for their ability to affect ligand binding to CTLA-4 in various contexts.

**Figure 1:**
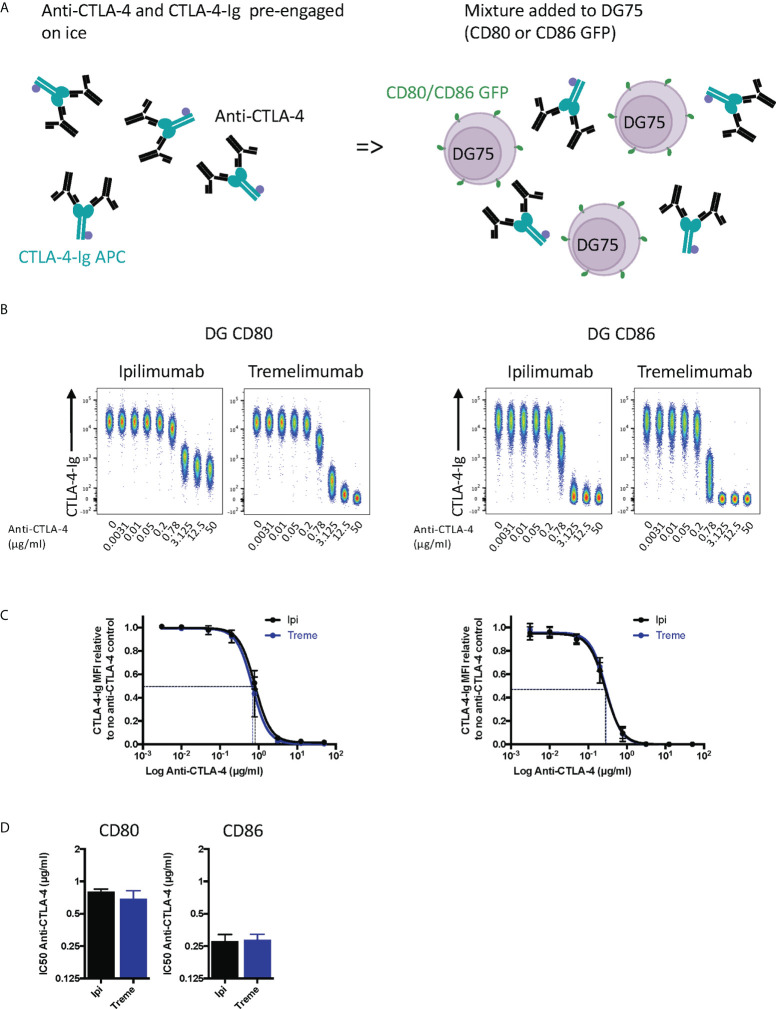
Pre-engagement of Ipilimumab and Tremelimumab with soluble CTLA-4-Ig prevents CTLA4 binding to cell bound CD80 or CD86 ligand. **(A)** Schematic of experimental set-up. A fixed dose (2µg/ml) of soluble APC conjugated Abatacept was incubated on ice with a titration of soluble anti-CTLA-4 Abs to pre-engage CTLA-4 with anti-CTLA-4. This was added to DG75 B cells expressing either CD80 or CD86-GFP and incubated on ice for 30 minutes. Cells were washed and analysed for Abatacept-ligand interaction by flow cytometry. All reagents were chilled on ice before use. **(B)** Representative concatenated FACS plots show impact of Abatacept-ligand engagement as the dose of anti-CTLA-4 Abs increased. **(C)** Anti-CTLA-4 doses were Log(x) transformed and dose response curves fitted using Prism v6 to obtain Log IC_50_ for ipilimumab(Ipi) (Black line) and tremelimumab(Treme) (Blue line). **(D)** Graphs show the mean IC_50_ with 95% confidence interval calculated using Prism v6. Data for ipilimumab and tremelimumab were acquired in separate 96-well plates and data presented as mean +/- SD from 3 independent experiments.

To establish the blocking potential of ipilimumab and tremelimumab we initially used a system where CD80 or CD86 ligands were expressed independently on a B cell line (DG75) and stained with fluorescently labelled soluble CTLA-4 (abatacept) allowing us to detect soluble CTLA-4–cellular ligand interactions. Initially, we performed this experiment by pre-binding anti-CTLA-4 to abatacept in solution and then exposing to ligand expressing cells **(** **Figure 1A** **).** If the antibodies bound to abatacept at the ligand binding site, it would be predicted that abatacept binding to DG75 cells would be impaired. We observed that in the absence of anti-CTLA-4, abatacept bound to both CD80 and CD86 expressing cells **(** **Figure 1B** **)**. Increasing doses of anti-CTLA-4 effectively inhibited the ability of abatacept to bind to ligand expressing cells, demonstrating the ability of anti-CTLA-4 to prevent CTLA-4-ligand interactions. We noted that when using CD80, ipilimumab did not completely block abatacept binding to background levels, compared to tremelimumab, which was more effective over the dose range used **(** **Figure 1B** **)**. These data appear in line with the slightly higher affinity of tremelimumab (16). It was also clear that the dose of anti-CTLA-4 required to prevent binding of abatacept to CD86 was lower than for CD80 (**Figures 1C, D**), in line with the weaker affinity of the CTLA-4 (abatacept)-CD86 interaction. As expected, isotype matched control antibodies did not inhibit abatacept staining demonstrating the specificity of ipilimumab and tremelimumab blockade **(** **Figure S1A**). Overall, despite subtle differences between the two CTLA-4 antibodies it was clear that both antibodies effectively prevented CTLA-4 ligand interactions when ligands were expressed on cells and performed in line with affinity expectations.

**Figure 2:**
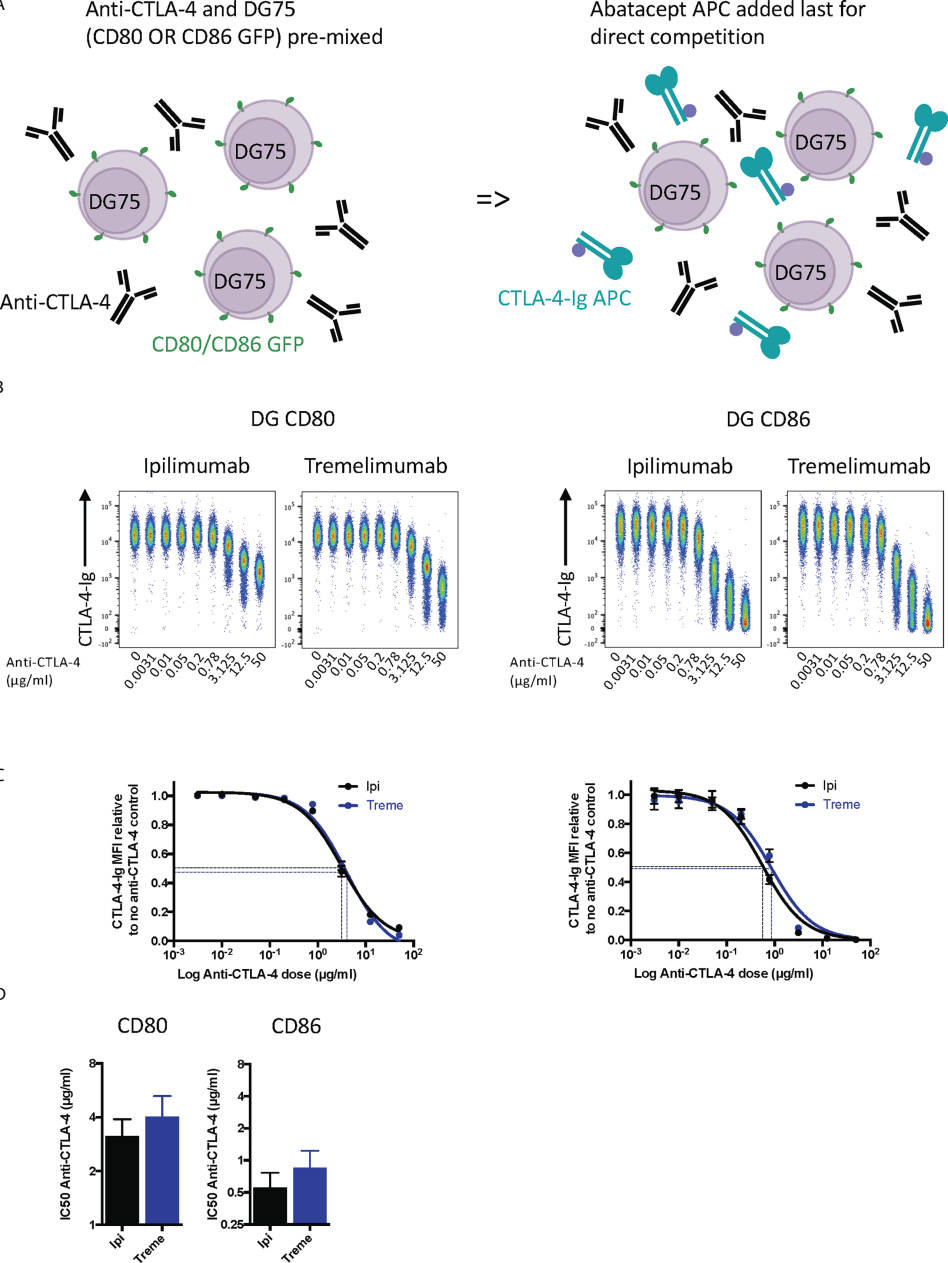
Ipilimumab and Tremelimumab outcompete cell bound CD80 or CD86 for engagement with soluble CTLA-4-Ig. **(A)** Schematic of experimental set-up. DG75 B cells expressing either CD80 or CD86-GFP were mixed with a titration of soluble anti-CTLA-4 Abs. A fixed dose (2µg/ml) of soluble APC conjugated Abatacept was added to assess direct competition between soluble anti-CTLA-4 and cell bound ligand for Abatacept binding. Cells were incubated on ice, washed and Abatacept-ligand engagement analysed by flow cytometry. All reagents/cells were chilled on ice before use. **(B)** Concatenated FACS plots show reduced Abatacept-ligand engagement as the dose of anti-CTLA-4 Abs increased. **(C)** Anti-CTLA-4 doses were Log(x) transformed and dose response curves fitted using Prism v6 to obtain Log IC_50_ for ipilimumab (Black line) and tremelimumab (Blue line). **(D)** Graphs show the mean IC_50_ with 95% confidence interval calculated using Prism v6. Data for ipilimumab and tremelimumab were acquired in separate 96-well plates and data presented as mean +/- SD from 3 independent experiments.

Whilst the above experiment established that both antibodies were clearly capable of blocking CTLA-4-ligand interactions in non-competitive settings, we performed a second experiment where anti-CTLA-4 and cell bound ligands directly competed for abatacept binding **(** **Figure 2A** **)**. These data provided similar results **(** **Figure 2B** **)** with the difference being that higher amounts of anti-CTLA-4 were now required to inhibit ligand–abatacept interactions with half maximal inhibition being ~4µg/ml for CD80 and 0.5µgml for CD86 **(** **Figures 2C, D** **)**. Once again, CD80-CTLA-4 interactions were more difficult to disrupt in line with affinity expectations of anti-CTLA-4 being more effective at disrupting the weaker CTLA-4 –CD86 interactions.

**Figure 3:**
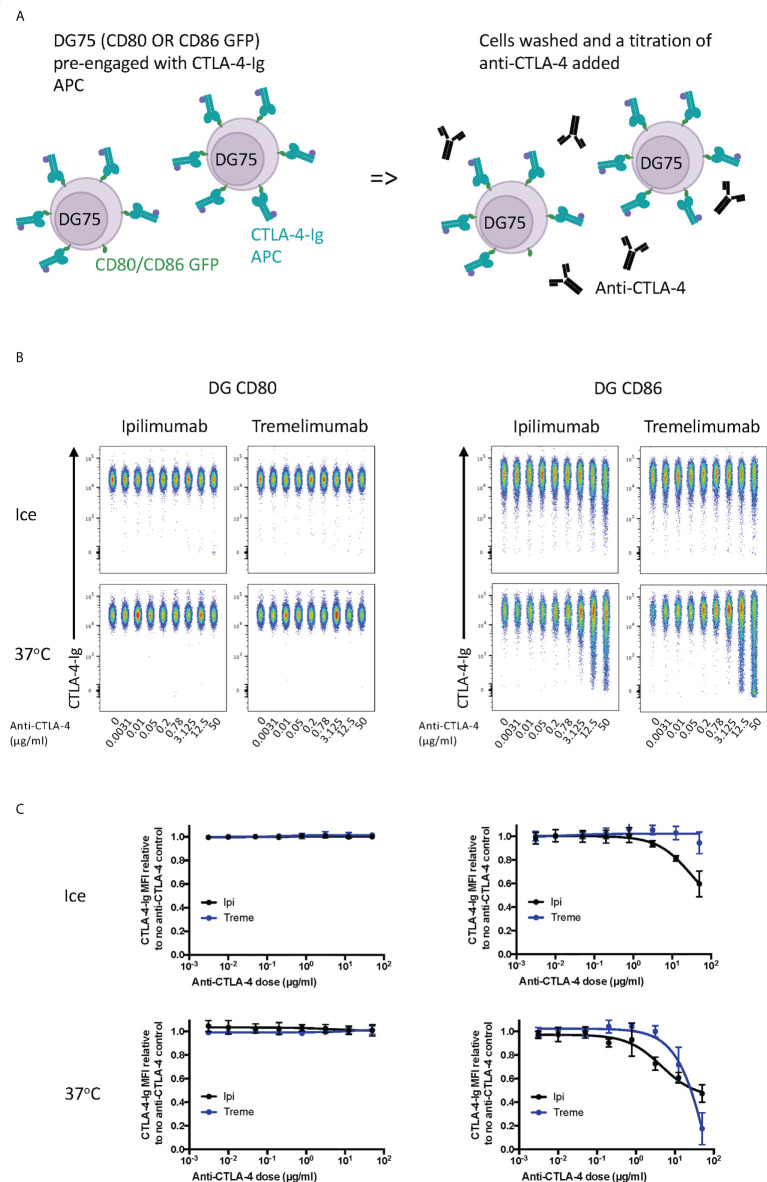
Ipilimumab and Tremelimumab are unable to displace pre-engaged CTLA-4-Ig from cell bound CD80 or CD86. **(A)** Schematic of experimental set-up. DG75 B cells expressing either CD80 or CD86-GFP were pre-incubated on ice with a fixed dose (2µg/ml) of soluble APC conjugated Abatacept to pre-engage soluble CTLA-4 with ligand. Cells were washed, treated with a titration of soluble anti-CTLA-4 Abs and incubated on ice or at 37°C. Cells were washed and analysed for Abatacept-ligand engagement by flow cytometry. All reagents/cells were chilled on ice before use. **(B)** Concatenated FACS plots show Abatacept-ligand binding at different doses of anti-CTLA-4 Abs when incubated on ice or when incubated at 37°C. **(C)** Dose response curves for ipilimumab (Black line) and tremelimumab (Blue line) presented as MFI CTLA-4-Ig binding from data shown in **(B)** Data presented are mean +/- SD from 3-6 independent experiments. Data for ipilimumab and tremelimumab were acquired in separate 96-well plates (n=3-6).

Finally, we tested the ability of ipilimumab and tremelimumab to displace abatacept using a membrane-bound ligand-receptor complex where cell-expressed ligands and abatacept binding was pre-established **(** **Figure 3A** **)**. This revealed that neither antibody could disrupt the high avidity CD80-CTLA-4 interaction over the time period tested **(** **Figures 3B, C** **)**, in line with the high avidity interaction between CTLA-4 and CD80. In contrast, we observed some impact on CTLA-4-CD86 interactions using ipilimumab this was observed only at high concentrations. Importantly, these data indicate that once ligand–CTLA-4 interactions were established the ability of antibody to effectively disrupt these interactions was limited, in particular for CD80. Given that such antibody-based displacement is likely to respond to temperature, we also repeated these experiments at 37°C to promote ligand-receptor dissociation. This again revealed that the interaction between CTLA-4 and CD80, once established, was not effectively disrupted by either antibody. In contrast, however, the weaker CTLA-4-CD86 interaction now showed significant disruption at higher concentrations of both CTLA-4 antibodies **(** **Figures 3B, C** **)**. Taken together, these data clearly demonstrated in 3 different scenarios that both ipilimumab and tremelimumab had robust blocking activity for soluble CTLA-4 binding to cell expressed ligands. However, once ligand-receptor complexes were established, the ability of antibodies to reverse these interactions was limited, with only CD86-CTLA-4 interactions being obviously affected.

**Figure 4:**
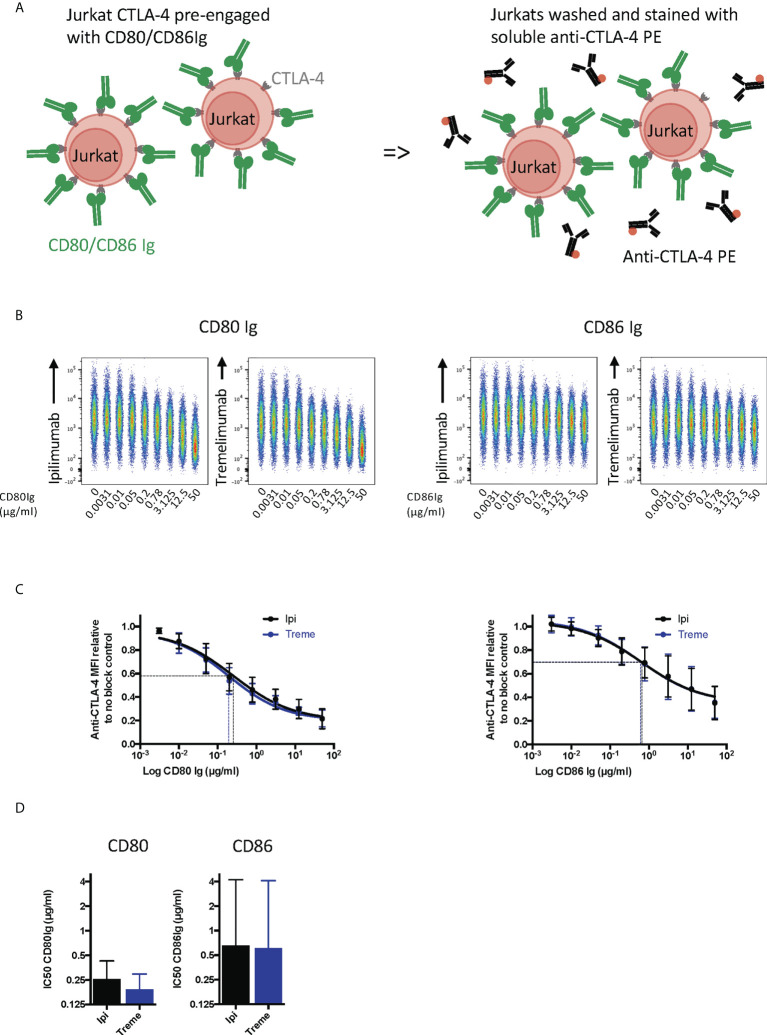
Pre-engagement of cell bound CTLA-4 with soluble CD80 and CD86Ig inhibits Ipilimumab and Tremelimumab from binding. **(A)** Schematic of experimental set-up. Jurkat cells expressing CTLA-4 were pre-incubated on ice with either soluble CD80 or CD86Ig to pre-engage CTLA-4 with ligand. Cells were washed, incubated with a fixed dose (2µg/ml) of soluble PE conjugated anti-CTLA4 Abs and incubated on ice. Cells were washed and analysed for anti-CTLA-4-CTLA-4 binding by flow cytometry. All reagents/cells were chilled on ice before use. **(B)** Concatenated FACS plots show reduced anti-CTLA-4 engagement as the dose of CD80 or CD86Ig was increased. CD80 or CD86Ig doses were Log(x) transformed and dose response curves fit using Prism v6 to obtain Log IC_50_ for ipilimumab (Black line) and tremelimumab (Blue line). **(C)** CD80 or CD86Ig doses were Log(x) transformed and dose response curves fitted using Prism v6 to obtain Log IC50 for ipilimumab (Black line) and tremelimumab (Blue line). **(D)** Graphs show the mean IC_50_ with 95% confidence interval calculated using Prism v6. Data for ipilimumab and tremelimumab were acquired in separate 96-well plates (n=3).

To establish whether anti-CTLA-4 blockade was affected when CTLA-4 (as opposed to ligand) was expressed on cells, we performed a series of experiments in reverse with CD80 and CD86-Ig being used to bind to cellular CTLA-4 **(** **Figure 4A** **).** This revealed a dose dependent inhibition of anti-CTLA-4 staining by soluble ligands when ligand was pre-engaged with CTLA4 **(** **Figure 4B** **).** As expected, the inhibition by CD86-Ig was less effective than CD80 with 50% inhibition requiring higher doses of CD86 to inhibit antibody binding due to its lower affinity **(** **Figures 4C, D** **).** However, both ligands interfered with the ability of ipilimumab and tremelimumab to bind to cellular CTLA-4 indicating that the antibodies recognise the CTLA-4 ligand binding site. Therefore, as expected, both anti-CTLA4 antibodies were inhibited by ligand-occupied CTLA-4. The specificity of antibodies for cellular CTLA-4 was clear since they did not bind to CTLA-4 deficient cells and staining CTLA-4 positive cells was only achieved with the appropriate primary and secondary antibodies **(** **Figures S1B, C** **)**.

**Figure 5:**
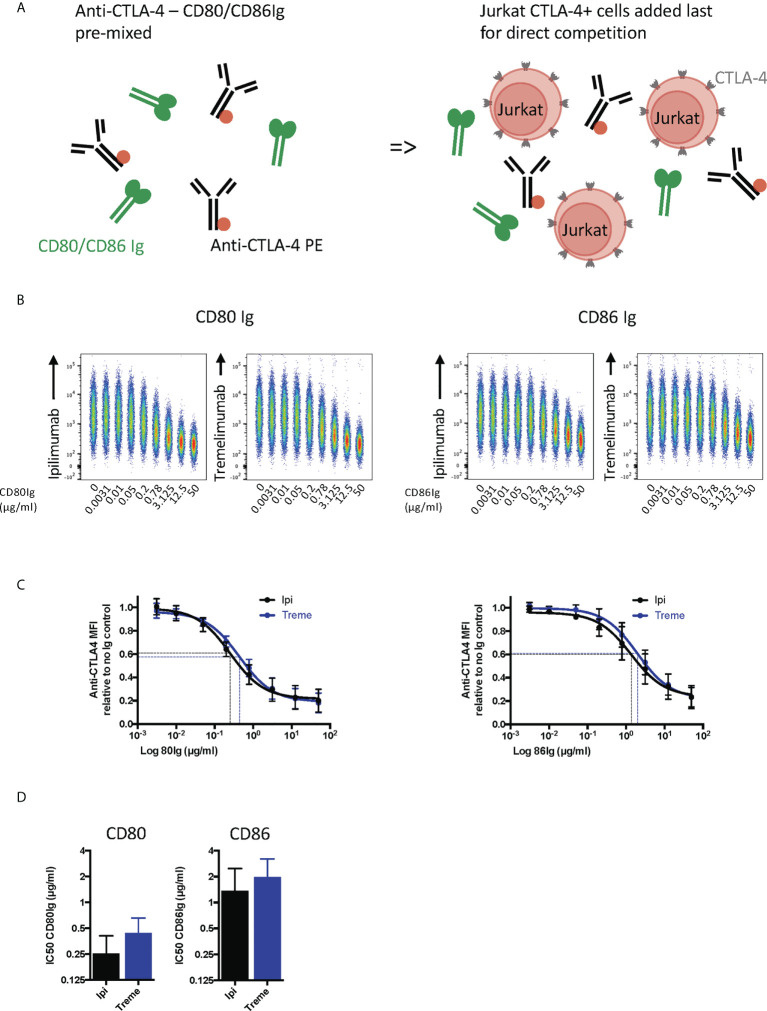
Soluble CD80 and CD86Ig outcompete Ipilimumab and Tremelimumab for engagement with cell bound CTLA4. **(A)** Schematic of experimental set-up. A fixed dose (2µg/ml) of soluble PE conjugated anti-CTLA4 Abs was pre-incubated with a titration of either soluble CD80 or CD86Ig on ice. Jurkat expressing CTLA-4 cells were added to facilitate direct competition between anti-CTLA4 Abs and CD80 or CD86Ig for cell bound CTLA-4 occupancy. Cells were incubated on ice, washed and anti-CTLA4-CTLA4 engagement analysed by flow cytometry. All reagents/cells were chilled on ice before use. **(B)** Concatenated FACS plots show reduced anti-CTLA-4-CTLA-4 engagement as the dose of CD80 or CD86Ig was increased. **(C)** CD80 or CD86Ig doses were Log(x) transformed and dose response curves fitted using Prism v6 to obtain Log IC_50_ for ipilimumab (Black line) and tremelimumab (Blue line). **(D)** Graphs show the mean IC_50_ with 95% confidence interval calculated using Prism v6. Data for ipilimumab and tremelimumab were acquired in separate 96-well plates (n=3).

We next repeated these experiments in a setting where soluble ligand and anti-CTLA-4 directly competed for CTLA-4 on Jurkat cells **(** **Figure 5A** **)**. This showed that CD80-Ig and CD86-Ig effectively interfered with anti-CTLA-4 binding in a dose-dependent manner **(** **Figure 5B** **)**. In line with affinity, CD80-Ig inhibited CTLA-4 binding at lower doses than the lower affinity CD86-Ig **(** **Figures 5C, D** **).** Nonetheless, at sufficient concentrations both ligands were able to inhibit the binding of anti-CTLA-4 to its target, indicating both ligands occupy binding sites overlapping with ipilimumab and tremelimumab and can compete with antibodies for binding.

**Figure 6:**
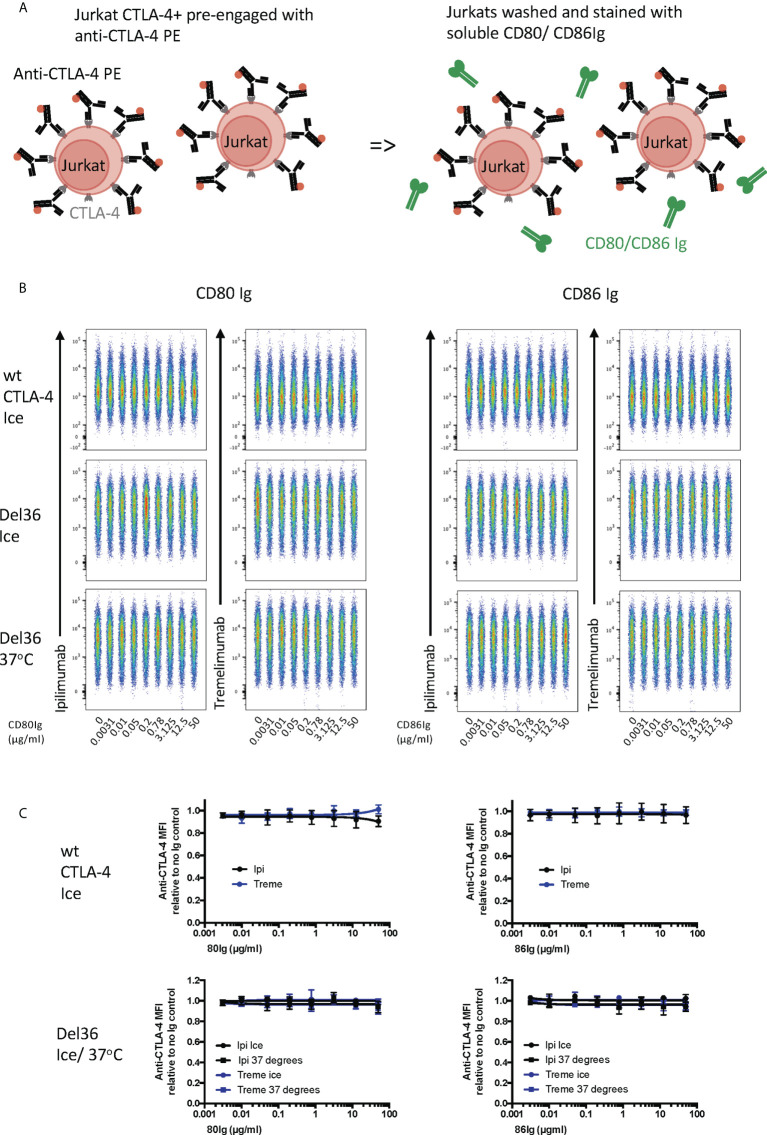
Soluble CD80 and CD86Ig are unable to displace pre-engaged Ipilimumab and Tremelimumab from cell bound CTLA-4. **(A)** Schematic of experimental set-up. Jurkat cells expressing CTLA-4 were pre-treated with a fixed dose (2µg/ml) of soluble PE conjugated anti-CTLA-4 Abs on ice to pre-engage anti-CTLA-4-CTLA-4. Cells were washed and a titration of either soluble CD80 or CD86Ig was added. Cells expressing WT CTLA-4 were incubated on ice and cells expressing a non-internalising CTLA-4 mutant (Del 36) were incubated at either on ice or at 37°C. Cells were washed and analysed for anti-CTLA-4-CTLA-4 staining by flow cytometry. **(B)** Concatenated FACS plots showinganti-CTLA-4 binding at various doses of CD80 or CD86Ig **(C)** Dose response curves for ipilimumab (Black line) and tremelimumab (Blue line) presented as MFI anti-CTLA-4 binding from data in **(B)** Data for ipilimumab and tremelimumab were acquired in separate 96-well plates (n=3-6).

Finally, we pre-bound either ipilimumab or tremelimumab to CTLA-4 expressed on Jurkat cells and then examined the ability of CD80- or CD86-Ig to displace these antibodies **(** **Figure 6A** **)**. As expected, high affinity CTLA-4 antibody binding completely blocked the ability of CTLA-4 to bind to either of its ligands at all ligand doses used **(** **Figures 6B, C** **)**. Moreover, since CTLA-4 is potentially capable of being internalised in these experiments, we also used a non internalising CTLA-4 cell line (Del 36) to determine if internalisation of CTLA-4 was responsible for these results. As shown in **Figures 6B, C**, pre binding of CTLA-4 antibodies to Del 36 and incubation at either 4°C or 37°C did not impact the ability of anti CTLA-4 to block ligand binding. This indicated that neither CD80 nor CD86 ligands were able to displace anti-CTLA4-CTLA4 interactions, once established.

Together the above data indicated that both anti-CTLA-4 antibodies had very similar abilities to disrupt CTLA-4-ligand interactions irrespective of whether ligand or receptor was in solution. Furthermore, receptor-ligand blockade performed in line with expectations based on interaction affinities, with the CD80-CTLA-4 high-avidity interaction proving more difficult to prevent. Of note however, it was clear that once pre-formed, ligand-receptor complexes could prevent binding of anti-CTLA-4 antibodies. This indicates that whilst ipilimumab and tremelimumab are effective blocking antibodies, for functional efficacy the antibodies need to interact with CTLA-4 before ligand binding is established.

### Ipilimumab and tremelimumab prevent CD80 and CD86 ligand loss by CTLA-4 transendocytosis

**Figure 7:**
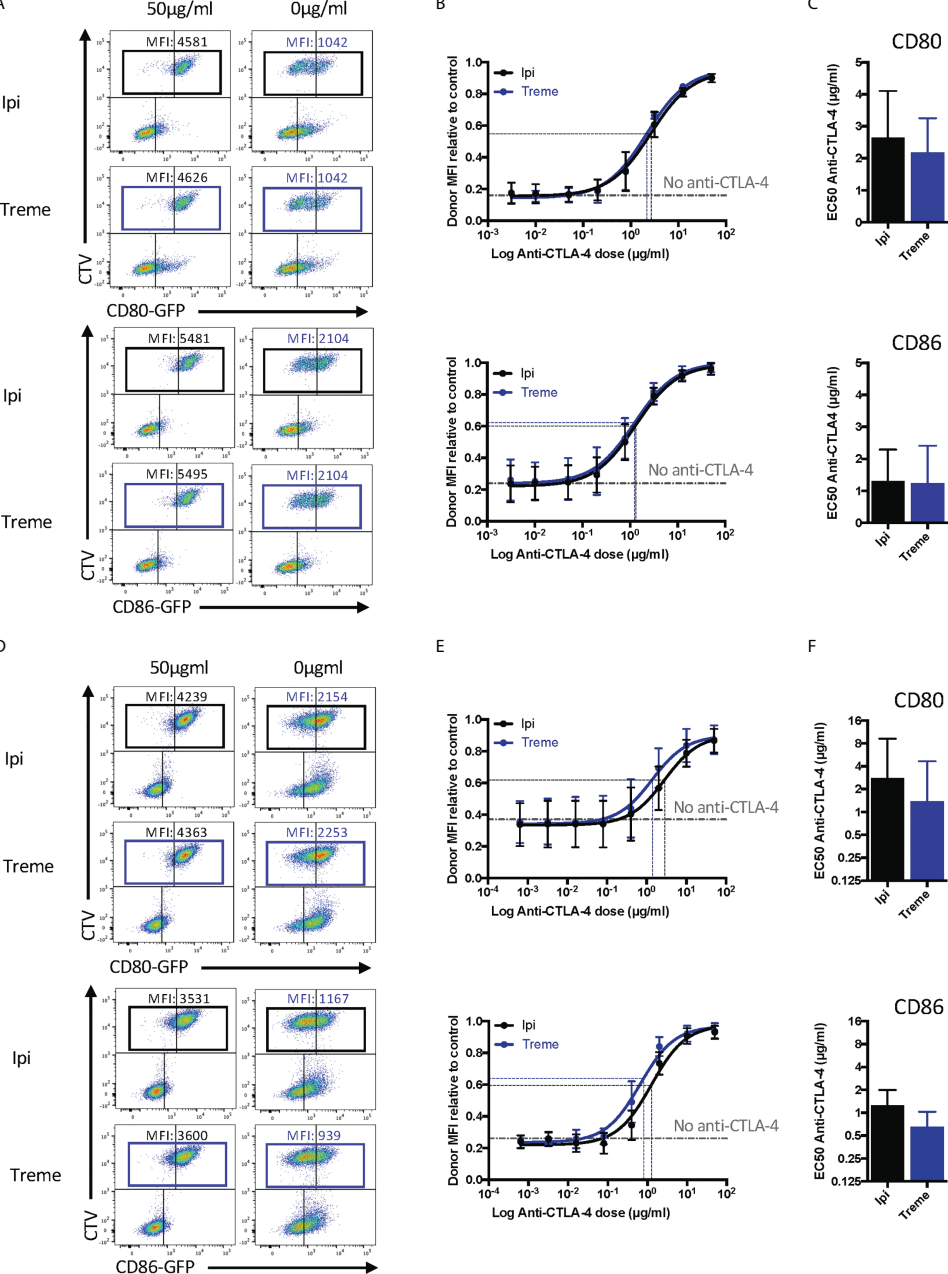
Ipilimumab and Tremelimumab block CD80 and CD86 ligand loss for APC by transendocytosis. **(A)** CTV stained CHO cells expressing either CD80 or CD86-GFP were cultured in the presence of CHO cells expressing CTLA-4 at a 1:1 ratio for 5 hours at 37°C. A titration of soluble ipilimumab (Black line) or tremelimumab (Blue line) were added at 0h. Percentage of CD80 or CD86-GFP ligand loss from CTV+ CHO cells by transendocytosis was determined by making the GFP MFI of CTV+ CHO cells relative to a negative control where no CTLA-4 was present. **(B)** Anti-CTLA-4 doses were Log(x) transformed and dose response curves fit using Prism v6 to obtain Log EC_50_ for Iipilimumab (Black line) and tremelimumab (Blue line). **(C)** Graphs show the mean EC_50_ with 95% confidence interval calculated using Prism v6. **(D-F)** As in A, B & C except with CTLA-4 expressing Jurkat and CTV stained DG75 B cells expressing CD80 or CD86-GFP. Data for ipilimumab and tremelimumab were acquired in separate 96-well plates, data presented as mean +/- SD from 3 independent experiments.

Having established that the CTLA-4 checkpoint antibodies used could effectively prevent CTLA-4-ligand interactions using soluble proteins, we wished to establish their impact on transendocytosis (13, 14). In this setting, cycling CTLA-4 protein captures and internalises CD80 and CD86 ligands from intercellular contacts between cells at 37°C. Accordingly, both ligand and receptor are in their normal membrane anchored state with appropriate dimerisation and make contact within a tightly opposed synapse between cells. We monitored ligand loss using a CHO cell system expressing CD80 or CD86 and mixed these with CTLA-4-expressing CHO cells to determine the impact of CTLA-4 blockade. As shown in **Figures 7A-C** these experiments revealed a clear dose-dependent blockade of ligand loss demonstrating that both ipilimumab and tremelimumab were highly effective at blocking CTLA-4-dependent ligand capture in completely cellular settings. Moreover, we observed no significant differences between these antibodies, which blocked both CD80 and CD86 transendocytosis effectively. In keeping with data from experiments with soluble proteins, the dose of antibody required to achieve 50% inhibition was greater for CD80 than for CD86.

Given that “immune synapses” may not be accurately reproduced between CHO cells expressing ligand and receptor, we also repeated these experiments using CD80 or CD86 expressing B cell lines and CTLA-4 expressing Jurkat T cells to assess any impact this might have. Once again, we observed **(** **Figures 7D-F** **)** very clear depletion of ligand in the absence of anti-CTLA-4, with dose-dependent antibody blockade of transendocytosis clearly evident. The dose response curve for CD80 blockade revealed the requirement for higher concentrations of antibody to disrupt transendocytosis compared to the CD86-CTLA-4. Thus, in two independent cell-cell systems, anti-CTLA-4 checkpoint antibodies completely ablated ligand downregulation by transendocytosis, which represents physiologically relevant CTLA-4-ligand interactions in a dynamic cellular setting. Once again, the features of antibody blockade continued to be in line with expectations based on ligand-receptor affinity.

### Ipilimumab and tremelimumab block ongoing transendocytosis of CD80 and CD86

**Figure 8:**
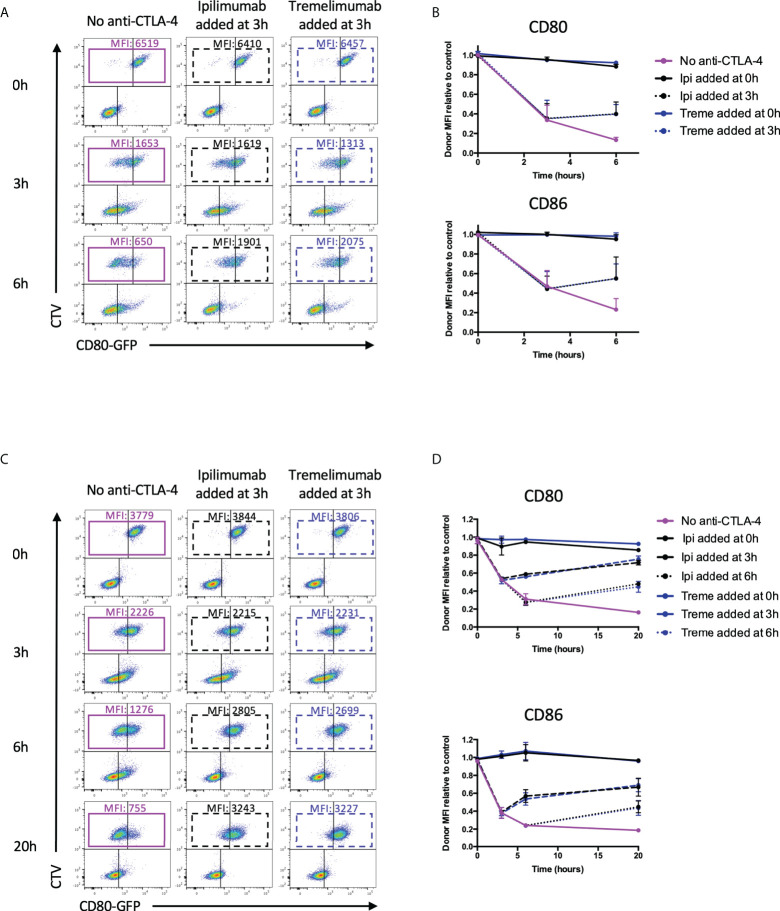
Ipilimumab and Tremelimumab interfere with pre-established transendocytosis of CD80 and CD86 to prevent further ligand loss from APC. **(A)** CTV stained CHO cells expressing either CD80 or CD86-GFP were cultured in the presence of CHO cells expressing CTLA-4 at a 1:1 ratio for 0, 3 or 6 hours at 37°C. Transendocytosis was established for 3 hours in the absence of anti-CTLA-4 Abs at which point soluble ipilimumab (dotted black line) or tremelimumab (dotted blue line) were spiked into culture. Transendocytosis was continued for a further 3 hours to observe the effect of anti-CTLA4 spike. Soluble anti-CTLA-4 antibodies were also added at timepoint 0h to block transendocytosis from the start of the assay (solid black – ipilimumab or blue – tremelimumab lines). Transendocytosis was also performed in the absence of anti-CTLA-4 treatment (Purple line). **(B)** % of CD80 or CD86-GFP ligand loss from CTV+ CHO cells by transendocytosis was determined by making the GFP MFI CTV+ CHO cells relative to a negative control where CTLA4 was not expressed (n=3). (**C**, **D**) As in A & B except with CTLA-4 expressing Jurkat and CTV stained DG75 B cells expressing CD80 or CD86-GFP cultured at a Jurkat:DG75 ratio of 2:1. Also an additional spike was performed at 6h and the assay was run for a total of 20h. Data for ipilimumab and tremelimumab were acquired in separate 96-well plates (n=2).

Since transendocytosis is a continuous, time-dependent process one concern is that the antibodies used in the above system may be able to prevent transendocytosis from initiating (by blocking CTLA-4 before cell contact is established) but may still fail to inhibit ongoing transendocytosis that has been pre-established. To address this issue, we repeated the above experiments, but added ipilimumab and tremelimumab either 3h or 6h after transendocytosis was initiated, i.e. once depletion of ligand had started. We then compared ligand downregulation after an additional period of transendocytosis. As shown in **Figure 8A**, in CHO cells we observed clear ligand downregulation in the absence of anti-CTLA-4, with time-dependent ligand loss increasing up to 6h (**Figure 8A** **-**Purple boxes). However, the addition of either tremelimumab or ipilimumab at 3h prevented further downregulation of ligand indicating that antibodies were capable of interrupting ongoing transendocytosis **(** **Figure 8B** **)**. As controls, the addition of anti-CTLA-4 at the beginning of the assay (0h) blocked ligand downregulation completely as expected. Once again, these findings were reproduced in T cell-B cell systems **(** **Figures 8C, D** **),** suggesting that neither the nature of the intercellular contact, nor the cell type affected the ability of these antibodies to inhibit this process. Moreover, these experiments indicated that after blockade, levels of ligand showed signs of recovery, indicating the ligand donor cells are clearly viable after transendocytosis. Overall, these data provided clear evidence that clinically used CTLA-4 checkpoint antibodies are able to prevent ligand-receptor interactions effectively in cellular settings.

## Discussion

CTLA-4 is well established as a key checkpoint that controls T cell activation and genetic defects lead to lymphoproliferation and profound autoimmunity in both humans and mice (3, 5, 18). Nonetheless, the mechanisms by which CTLA-4 functions have been the source of debate. Predominantly expressed on activated T cells and Treg, evidence suggests that a major function of CTLA-4 is *via* its expression on Treg (19–21). We have shown that CTLA-4 can operate *via* controlling access of CD28 to their shared ligands either by competition for ligand binding, or *via* the physical removal of ligands due to transendocytosis (13). In either setting the blockade of CTLA-4 access to its ligands is predicted to inhibit CTLA-4 function. An alternate possibility is that the function of anti-CTLA-4 in tumour therapy may be to induce the Fc-mediated destruction of Treg cells within tumour sites (11, 12). Fc-mediated depletion of Treg *via* CTLA-4 targeting by macrophages would not strictly require blockade of CTLA-4-ligand interactions and could therefore work using non-blocking antibodies to CTLA-4, that can provide an Fc region for phagocyte targeting.

Recently it was reported that use of anti-CTLA-4 antibodies such as ipilimumab were unable to block ligand binding in certain settings, particularly where ligand was cell expressed or immobilised and in the setting of transendocytosis (15). Given that structural data indicates that ipilimumab binds in the ligand binding site (16), this suggests that the biological context of ligand receptor interactions may influence blocking capability. Given that antibodies lacking blocking activity could retain efficacy and may have reduced side effects this has prompted a revisiting of the fundamental activity of currently used anti-CTLA-4 treatments. Our experiments showed that both ipilimumab and tremelimumab were capable of robust blockade of CTLA-4-ligand interactions. Moreover, all blockade performed in line with expectations based on their reported intrinsic affinities (22), where CD80-CTLA-4 interactions were consistently more difficult to block. As expected, blockade of CD86-CTLA-4 interactions was achieved at lower doses, suggesting that CD80 ligands may be more readily available than CD86 at a sub optimal doses of antibody, which may occur during therapy. A paradox in anti-CTLA4 therapy is that blocking CTLA-4 should lead to excess CD28 co-stimulation, which could lead to increased Treg expansion as has been reported *in vivo* (23) and as we have shown *in vitro* (24). Whilst CTLA4 suppressive function will be blocked other Treg suppressive mechanisms may still function, potentially affecting outcomes in anti-tumour immunity. Given that CD86 is the main driver of Treg expansion and homeostasis^23^, the ability of checkpoint antibodies to control CTLA-4-CD86 interactions may be important.

In contrast to a previous report (15), we did not observe any differential blocking effects on CTLA-4/ligand interactions regardless of whether the ligands were soluble or cell expressed. Moreover, in transendocytosis assays, where both ligand and receptor are expressed in a cellular context, we found that CTLA-4 antibodies could effectively block ligand removal from cell-cell contacts. Our observations are therefore in contrast to the findings of Du et al. (15). One possibility raised by our observations is that the impact of ipilimumab and tremelimumab could depend substantially on whether ligands were pre-engaged with CTLA-4. Accordingly, pre-binding between CTLA-4 and ligand prevented anti-CTLA-4 from binding, with little sign that the antibody could displace ligands, once complexes were formed. This is in line with the location of ligand binding overlapping with that of the antibodies (16) and therefore some of the findings of Du et. al., could relate to the timing of reagent addition. Du et al., also tested some interactions in ELISA assays, which we did not use here, raising the possibility the differences in our findings could relate to the type of assay performed. However, they went on to suggest that ipilimumab did not prevent CTLA-4-Ig binding to CD80 in cellular assays similar to those used here. As far as can be ascertained, the binding of CTLA-4-Ig to either the ligand expressing cell or to the anti-CTLA-4 antibody took place in direct competition as per our experiments in **Figure 2**. It is therefore hard to find an explanation that reconciles our two sets of data from these apparently similar experiments.

Our results from transendocytosis assays also differ from those of Du et al. (15), in that we find anti-CTLA-4 antibodies blocked both ligand downregulation from the donor cell and ligand uptake by CTLA-4 expressing cells our assays. Indeed, downregulation of ligand from donor APCs could be to some extent reversed even once transendocytosis was established, indicating that antibody blockade was effective even once cell contacts were robustly established. In these assays, Du et.al., appear to have used anti-CTLA-4 Fab fragments whereas we use intact IgG antibodies. However, in principle this should not impact the ability of the antibody fragments to block ligand receptor interactions. There are also some differences in the way we perform and analyse transendocytosis assays by flow cytometry. In particular, we use cell trace violet as a marker of ligand donor cells, allowing us to gate accurately on donor or recipient populations (see **Figure S2** **)**. In this way we can ensure that ligand acquisition by CTLA-4 cells is not the result of donor cells being stuck to CTLA-4 cells and we can also identify donor cells even if all GFP-Ligand has been removed. The assays used by Du et.al, differ and may be more prone to cell conjugate inclusion, (despite scatter gating) and appear to measure ligand acquisition by CTLA-4 cells, rather than ligand loss from donor cells as used here. The latter is a more reliable indicator of transendocytosis in our experience. Nonetheless, despite these technical differences between our studies there is no obvious explanation for our different conclusions regarding the blocking capabilities of CTLA-4 checkpoint antibodies.

Importantly, for CTLA-4 antibodies to block therapeutically our data suggest that CTLA-4 must be targeted before ligand binding, particularly in respect of CD80, which forms a highly avid dimer-dimer complex with CTLA-4 (25). This is particularly significant in a transendocytosis setting where CTLA-4 is recycled from intracellular compartments (26). However, our data indicate it is still possible for anti-CTLA-4 to target CTLA-4 before ligand binding even during the transendocytosis process. In particular, this is indicated by the fact that both ipilimumab and tremelimumab could clearly inhibit the high affinity CD80 interaction during transendocytosis. Surprisingly, we also found that even once transendocytosis is fully established antibodies could inhibit further ligand uptake, suggesting these antibodies gain access to zones of membrane contact, or bind to CTLA-4 before this point. Overall, we did not identify settings where the blocking capabilities of ipilimumab or tremelimumab were compromised and observed they performed as predicted by biophysical data.

Finally, we found that the two checkpoint antibodies tested had similar activity in our assays. These data are in keeping with structural data showing similar binding sites for both antibodies, which overlap on CTLA-4 and occupy the ligand binding site. Therefore, our data establish that both clinically utilised anti-CTLA-4 antibodies (ipilimumab and tremelimumab) have similar properties and effectively block CTLA-4-ligand interactions in a variety of settings.

## Data availability statement

The original contributions presented in the study are included in the article/**Supplementary Material**, further inquiries can be directed to the corresponding authors.

## Author contributions

CW, AK and MR designed and performed experiments and analysed the data. SD,CL and DS conceptualised, supervised and obtained funding for the work. DS and CW wrote the draft manuscript and all authors reviewed and edited the manuscript. All authors contributed to the article and approved the submitted version.

## Funding

CW was funded by a research grant from AstraZeneca to DS. AK was funded by Wellcome. MR is funded by an MRC industrial CASE award with AstraZeneca. DS is a recipient of a Wellcome Investigator Award(204798/Z/16). This research was funded in whole, or in part, by the Wellcome Trust (Grant 204798/Z/16). For the purpose of Open Access, the author has applied a CC BY public copyright license to any Author Accepted Manuscript version arising from this submission.

## Conflict of interest

Authors CL and SD are full time employees and shareholders of AstraZeneca.

The remaining authors declare that the research was conducted in the absence of any commercial or financial relationships that could be construed as a potential conflict of interest.

The authors declare that this study received funding from AstraZeneca. The funder had the following involvement in the study: Co-supervision of the project, provision of reagents and writing of the manuscript.

## Publisher’s note

All claims expressed in this article are solely those of the authors and do not necessarily represent those of their affiliated organizations, or those of the publisher, the editors and the reviewers. Any product that may be evaluated in this article, or claim that may be made by its manufacturer, is not guaranteed or endorsed by the publisher.

## Supplementary material

Supplementary Figure 1 Specificity of Anti-CTLA4 antibodies. **(A)** CD80 or CD86-GFP expressing DG75 B cells were mixed with 50µg/ml anti-CTLA4 Abs or isotype controls. 2µg/ml APC-conjugated abatacept was added and incubated on ice for 30 minutes. LH panel shows representative FACS plots and RH panels show aggregate data for abatacept binding (MFI) in the presence of ipilimumab(Ipi) or tremelimumab(Treme) or an isotype control from 3 independent experiments. **(B)** CTLA4 expressing or non-expressing Jurkat cells were stained with anti-CTLA-4 Abs or isotype controls for 30 minutes on ice. Cells were washed and stained with an anti-human secondary Ab for 30 minutes on ice. LH panel shows representative FACS plots and RH panels show aggregate data for CTLA-4 binding (MFI) from3 independent experiments. **(C)** CTLA4 expressing or non-expressing Jurkat cells were stained directly with PE-conjugated anti-CTLA-4 Abs on ice for 30 minutes and analysed by flow cytometry. LH panel shows representative FACS plots and RH panels show aggregate data for CTLA-4 binding (MFI) from >10 independent experiments.

Supplementary Figure 2 Gating strategy for transendocytosis assays. Mixtures of Jurkat cells and CTV-labelled DG75 B cells expressing GFP ligands were gated by scatter and then on singlet cells. Cells were then gated for analysis into CTV+ (ligand donor cells) and CTV-ve (CTLA-4 + cells) to determine GFP ligand loss from the CTV+ donors and GFP gain by CTLA-4 + Jurkat recipient cells.
