# Supplementary figures and images for: Impact of CTLA-4 checkpoint antibodies on ligand binding and Transendocytosis
Source: Front Immunol. 2022 Aug 31;13:871802. doi: 10.3389/fimmu.2022.871802 (PMC9471429; doi:10.3389/fimmu.2022.871802)

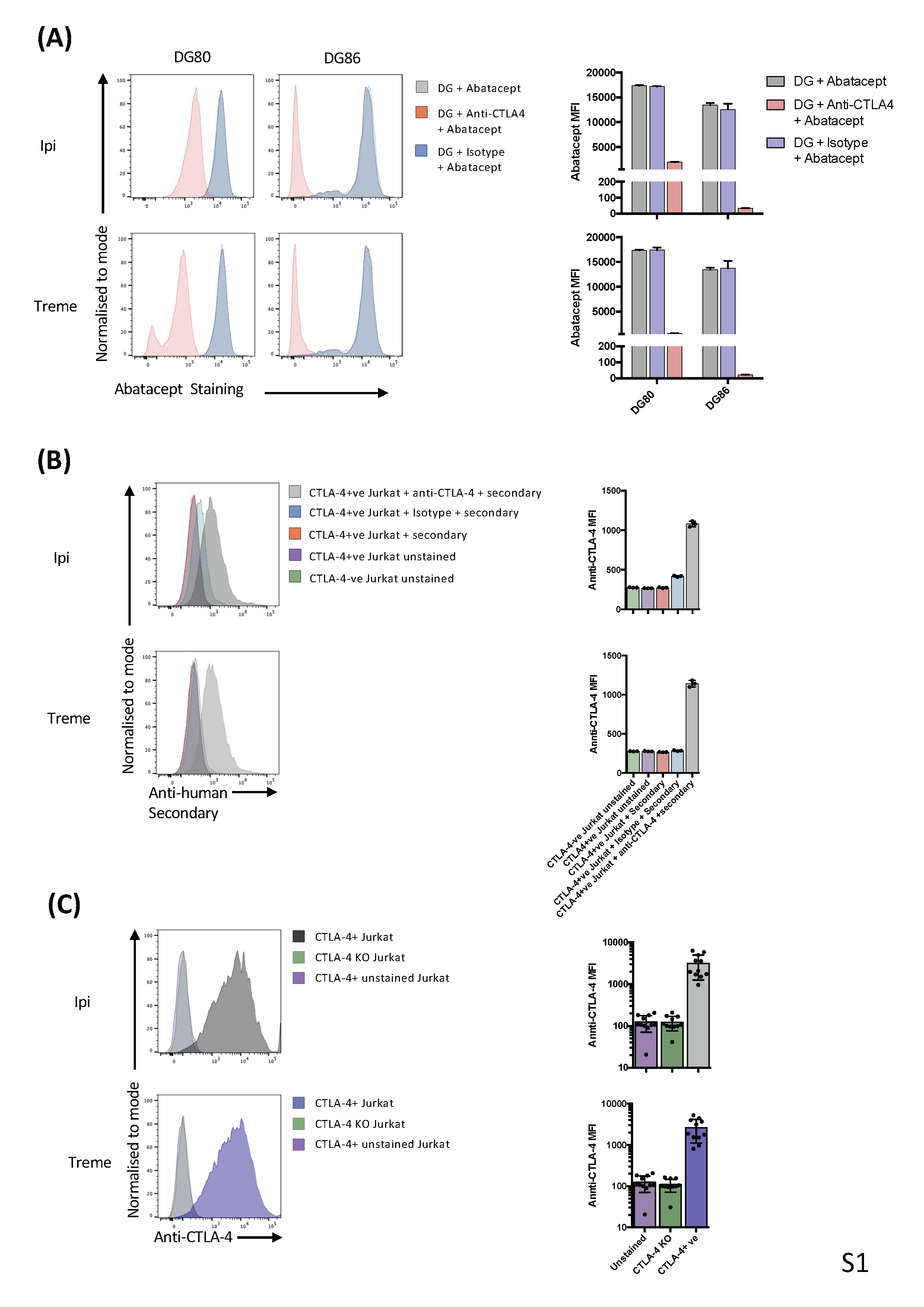

Supplement: Supplementary Figure 1 — Specificity of Anti-CTLA4 antibodies. (A) CD80 or CD86-GFP expressing DG75 B cells were mixed with 50µg/ml anti-CTLA4 Abs or isotype controls. 2µg/ml APC-conjugated abatacept was added and incubated on ice for 30 minutes. LH panel shows representative FACS plots and RH panels show aggregate data for abatacept binding (MFI) in the presence of ipilimumab(Ipi) or tremelimumab(Treme) or an isotype control from 3 independent experiments. (B) CTLA4 expressing or non-expressing Jurkat cells were stained with anti-CTLA-4 Abs or isotype controls for 30 minutes on ice. Cells were washed and stained with an anti-human secondary Ab for 30 minutes on ice. LH panel shows representative FACS plots and RH panels show aggregate data for CTLA-4 binding (MFI) from3 independent experiments. (C) CTLA4 expressing or non-expressing Jurkat cells were stained directly with PE-conjugated anti-CTLA-4 Abs on ice for 30 minutes and analysed by flow cytometry. LH panel shows representative FACS plots and RH panels show aggregate data for CTLA-4 binding (MFI) from >10 independent experiments. [file Image_1.tiff]

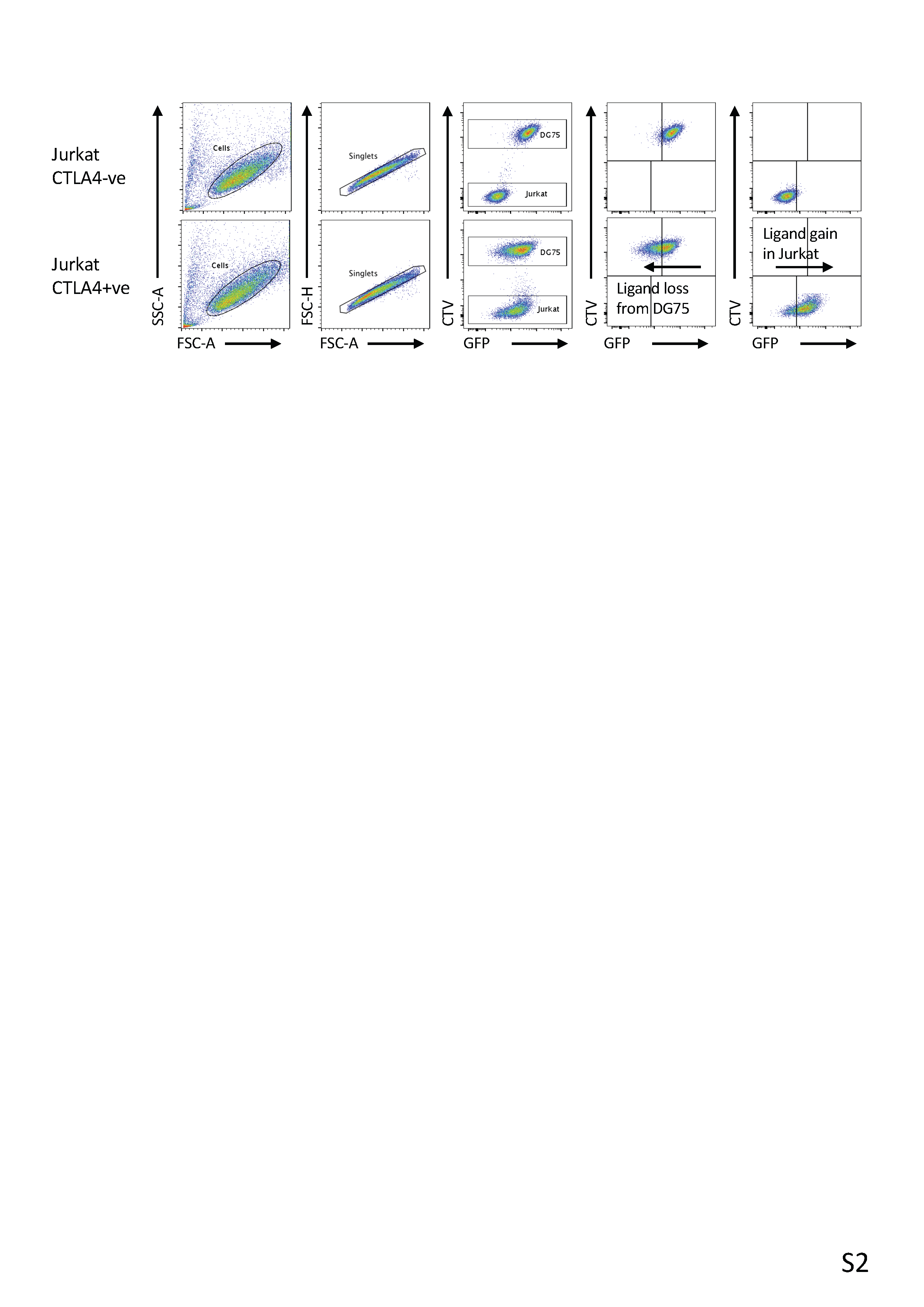

Supplement: Supplementary Figure 2 — Gating strategy for transendocytosis assays. Mixtures of Jurkat cells and CTV-labelled DG75 B cells expressing GFP ligands were gated by scatter and then on singlet cells. Cells were then gated for analysis into CTV+ (ligand donor cells) and CTV-ve (CTLA-4 + cells) to determine GFP ligand loss from the CTV+ donors and GFP gain by CTLA-4 + Jurkat recipient cells. [file Image_2.tiff]
